# Added value of histogram analysis of ADC in predicting radiation-induced temporal lobe injury of patients with nasopharyngeal carcinoma treated by intensity-modulated radiotherapy

**DOI:** 10.1186/s13244-022-01338-w

**Published:** 2022-12-17

**Authors:** Dan Bao, Yanfeng Zhao, Wenli Wu, Hongxia Zhong, Meng Yuan, Lin Li, Meng Lin, Xinming Zhao, Dehong Luo

**Affiliations:** 1 Department of Radiology, National Cancer Center/National Clinical Research Center for Cancer/Cancer Hospital, Chinese Academy of Medical Sciences and Peking Union Medical College, 17 Panjiayuan Nanli, Chaoyang District, Beijing, 100021 China; 2 Medical Imaging Center, Liaocheng Tumor Hospital, Shandong, 252000 China; 3 Department of Radiation Oncology, National Cancer Center/National Clinical Research Center for Cancer/Cancer Hospital, Chinese Academy of Medical Sciences and Peking Union Medical College, 17 Panjiayuan Nanli, Chaoyang District, Beijing, 100021 China; 4 Department of Radiology, National Cancer Center/National Clinical Research Center for Cancer/Cancer Hospital & Shenzhen Hospital, Chinese Academy of Medical Sciences and Peking Union Medical College, Shenzhen, 518116 China

**Keywords:** Nasopharyngeal carcinoma, Diffusion-weighted MRI, Temporal lobe, Radiation therapy, Radiomics

## Abstract

**Background:**

This study evaluated the predictive potential of histogram analysis derived from apparent diffusion coefficient (ADC) maps in radiation-induced temporal lobe injury (RTLI) of nasopharyngeal carcinoma (NPC) after intensity-modulated radiotherapy (IMRT).

**Results:**

Pretreatment diffusion-weighted imaging (DWI) of the temporal lobes of 214 patients with NPC was retrospectively analyzed to obtain ADC histogram parameters. Of the 18 histogram parameters derived from ADC maps, 7 statistically significant variables in the univariate analysis were included in the multivariate logistic regression analysis. The final best prediction model selected by backward stepwise elimination with Akaike information criteria as the stopping rule included kurtosis, maximum energy, range, and total energy. A Rad-score was established by combining the four variables, and it provided areas under the curve (AUCs) of 0.95 (95% confidence interval [CI] 0.91–0.98) and 0.89 (95% CI 0.81–0.97) in the training and validation cohorts, respectively. The combined model, integrating the Rad-score with the T stage (*p* = 0.02), showed a favorable prediction performance in the training and validation cohorts (AUC = 0.96 and 0.87, respectively). The calibration curves showed a good agreement between the predicted and actual RTLI occurrences.

**Conclusions:**

Pretreatment histogram analysis of ADC maps and their combination with the T stage showed a satisfactory ability to predict RTLI in NPC after IMRT.

**Supplementary Information:**

The online version contains supplementary material available at 10.1186/s13244-022-01338-w.

## Key points

- Histogram parameters from pretreatment ADC maps are associated with RTLI in NPC.
- The ADC and combined models show better performance than the T stage alone.
- The combined model shows excellent performance in predicting RTLI in different subgroups.

**Supplementary Information:**

The online version contains supplementary material available at 10.1186/s13244-022-01338-w.

## Background

Radiotherapy remains the primary treatment for nasopharyngeal carcinoma (NPC) [1]. However, radiation-induced temporal lobe injury (RTLI) can be a serious complication that severely affects the quality of life and long-term prognosis [2, 3]. Although radiotherapy techniques with better conformance, such as intensity-modulated radiotherapy (IMRT), provide better long-term disease control and are less toxic [4], RTLI is still reported in 4.6–8.5% of patients [5, 6]. Symptom-based diagnosis of RTLI is problematic in clinical practice because most patients are asymptomatic even at a very late stage or are already in a stage of non-reversible deterioration when noticeable symptoms start to appear, during which treatment has limited effect [7]. In contrast, if RTLI could be identified early or even predicted before the onset of symptoms, personalized intervention could be provided in advance to reverse the unfavorable situation. Therefore, it is particularly important to predict RTLI noninvasively after IMRT.

Several recent studies have focused on predicting RTLI in patients with NPC [8–10]. Studies based on radiation dosimetry-related factors have shown some predictive potential [9–11]; however, the optimal dose/volume predictors of RTLI still vary among different studies, and clinical applications are limited [12]. The imaging diagnosis of RTLI mainly depends on magnetic resonance imaging (MRI) findings. MRI is a suitable tool with multiple techniques available, which not only enables structure depiction, but also function quantification.

Diffusion-weighted imaging (DWI) is a functional technique that provides information about the tissue microenvironment depending on the microscopic mobility of water [13]. Because of the Brownian motion of water molecules, DWI can be quantified using the apparent diffusion coefficient (ADC) derived from the Gaussian diffusion model. DWI and dynamic contrast-enhanced MRI showed potential for predicting the response to radiation therapy for head and neck paragangliomas [14], promise in differentiating head and neck schwannomas and paragangliomas [15], detecting occult primary head and neck squamous cell carcinoma [16], and survival prediction in patients with head and neck squamous cell carcinoma treated with (chemo)radiation [17]. Diffusion tensor imaging (DTI) and DWI can be used to differentiate benign and malignant head and neck lesions [18]. DWI and ADC images can also be used for segmentation [19]. Histogram analysis is a mathematical method that provides information about the distribution of data in the selected region of interest (ROI), providing more information that is often ignored by the human eye [20]. To the best of our knowledge, only a few studies have investigated the association between MRI features and RTLI occurrence in NPC patients [21–25]. Moreover, histogram analysis has not been extensively explored in this field.

This study aimed to investigate the value of pretreatment histogram analysis of ADC in the prediction of RTLI in patients with NPC.

## Methods

### Patients

**Fig. 1:**
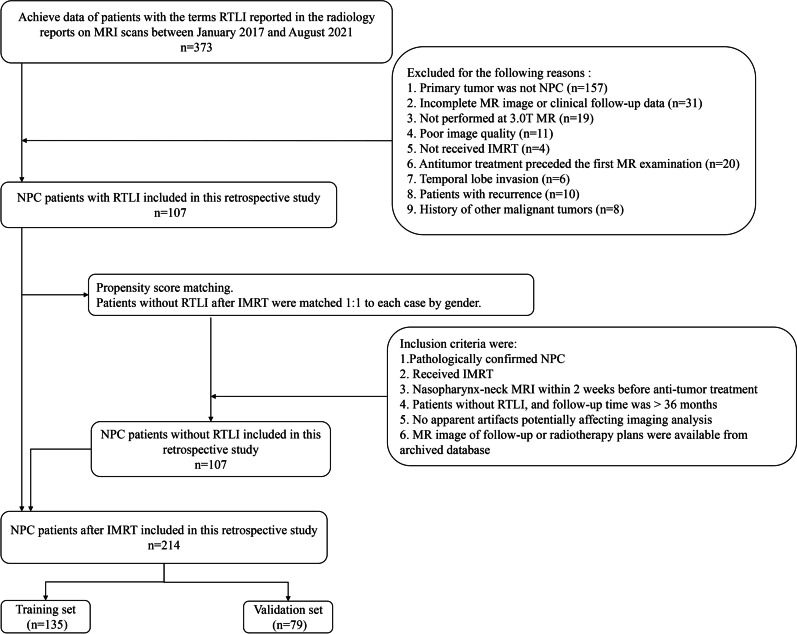
Diagram for inclusion of patients into the study. IMRT = intensity-modulated radiotherapy, NPC = nasopharyngeal carcinoma, RTLI = radiation-induced temporal lobe injury

This retrospective single-center study was approved by the local institutional review board, which waived the requirement for informed consent. Our radiological database was queried between January 2017 and December 2021. The inclusion criteria were as follows: (a) histopathologically confirmed NPC; (b) head–neck MRI including DWI performed in our institution within 2 weeks before any treatment; (c) receiving IMRT; and (d) RTLI after IMRT was found during follow-up. The exclusion criteria were as follows: (a) history of any prior local–regional therapies, (b) poor image quality due to severe artifacts, (c) temporal lobe invasion, and (d) recurrent NPC. In total, 107 patients with RTLI were included according to the inclusion and exclusion criteria. Propensity score matching was performed for this cohort of patients. The control group included patients without RTLI after IMRT who were matched 1:1 to each case by sex (Fig. 1). Thus, 214 patients were included in this study, who were randomly allocated to a training set (135 patients) and a validation set (79 patients) at a ratio of 6:4.

### Clinical data

Clinical information was analyzed in this study, including sex, age, neutrophil-to-lymphocyte ratios, TNM stage, pathologic subtype, treatment regimen, date of pretreatment MRI scan, dosimetric parameters including maximum dose for each temporal lobe, and planning gross tumor volume including the primary nasopharyngeal tumor or enlarged retropharyngeal nodes.

### Treatment regimen and follow-up

All patients underwent a standard treatment regimen consisting of IMRT and concurrent or adjuvant chemotherapy, with or without induction chemotherapy, based on the National Comprehensive Cancer Network guidelines [26]. All patients were treated with IMRT using the HiArt TomoTherapy system (Accuray, Sunnyvale, CA) or a Varian-600CD linear accelerator (Varian Medical Systems, Palo Alto, CA) with a prescribed dose of 70–74 Gy in 30–33 fractions [27].

After radiation therapy, patients routinely underwent follow-up MRI every 1–3 months during the first 2 years, every 6 months in years 3–5, and annually thereafter. The endpoint of this study was the development of RTLI or the last follow-up for non-RTLI (> 36 months).

### Diagnostic criteria of RTLI

The diagnostic criteria for temporal lobe injury (TLI) were as follows [28]: (a) white matter lesions with homogeneous high signal intensity on T2-weighted images and low signal intensity on T1-weighted images without contrast enhancement, (b) contrast-enhanced lesions with or without necrosis on post-contrast T1-weighted images with heterogeneous signal abnormalities on T2-weighted images, and (c) cysts or round or oval well-defined lesions with very high signal intensity on T2-weighted images with a thin or imperceptible wall.

### Image acquisition

All patients were examined using a 3.0-T MR scanner (GE Discovery MR 750; GE Healthcare, Chicago, IL) with an 8-channel head and neck phased array coil. DWI-MRI examinations were acquired axially using a single-shot echo-planar imaging technique with a spectral pre-saturation attenuated inversion-recovery fat-suppressed pulse sequence (repetition time/echo time, 4000/51 ms; bandwidth, 250 kHz; field of view, 24 cm; slice thickness, 5 mm; slice gap, 1 mm; number of excitations, 6.0). Diffusion gradients were applied with b values of 0 and 800 s/mm^2^.

### Temporal lobe segmentation

ADC maps were automatically calculated from b0 and b800 images using the MRI console. MRI images were reviewed by two radiologists (with 18 and 5 years of experience in head and neck imaging, respectively). A temporal lobe ROI was drawn on the *b* = 800 s/mm^2^ DWI of the pretreatment MRI using ITK-SNAP (version 3.6.0-RCI; http://www.itk-snap.org). The ROI was manually delineated along the boundaries of the middle and lower portions of the bilateral temporal lobes from the top level of the cerebral peduncle to the bottom of the temporal lobe (Additional file 1: Figure S1). One junior radiologist (Dan Bao) manually delineated and a senior neuroradiologist (Yanfeng Zhao) verified that both were blinded to clinical outcomes. The ROIs were then propagated to ADC maps. Inter-observer segmentation variability was evaluated using the Dice similarity coefficient (DSC) [29] in 50 randomly selected patients.

### Histogram analysis

Quantitative analysis was performed by a radiologist with five years of experience in head and neck MRI. For quantitative analysis, all ROIs were merged into the volume of interest in the ADC maps. Histogram features were extracted using the non-open source software Analysis Kit (Version v3.0.1. A, GE Healthcare) with the following parameters: skewness, kurtosis, entropy, energy, range, uniformity, mean, median, minimum, maximum, variance, 10th percentile, 90th percentile, interquartile range (IQR), mean absolute deviation, robust mean absolute deviation, root mean square, and total energy.

### Development and validation groups

The training cohort used 60% of the dataset and the validation cohort used the remaining 40%. Univariate and multivariate logistic regression analyses were performed using the training data to determine the predictive factors for RTLI. The backward stepwise was used to select variables included in the best models, and the Akaike’s information as the stopping criterion [30, 31]. A function based on the variance inflation factor was used to check the collinearity of the variables included in the regression equation, with a variance inflation factor greater than 10 indicating multicollinearity [32]. Receiver operating characteristic curve (ROC) analyses of significant findings and combined analyses were performed to evaluate the predictive performance. Sensitivity, specificity, negative predictive value, and positive predictive value with 95% confidence intervals (CIs) were calculated. The areas under the curve (AUC) were compared using the DeLong method.

According to the results of the multivariate analysis, the predictive model was visualized as a nomogram to stratify the individual risk of RTLI. A calibration curve was used to describe the agreement between predicted and observed RTLI occurrence probabilities. The Hosmer–Lemeshow test was performed to explain the goodness-of-fit of the multivariate logistic model. Decision curve analysis (DCA) was used to evaluate the clinical usefulness by quantifying the net benefits of the predictive model in the validation set. The optimum cutoff value of the signature was identified using ROC analysis based on its association with the RTLI outcome. Accordingly, the patients were divided into low- and high-risk groups, for which the RTLI predictive outcomes were compared by ROC analysis in subgroups within clinical–pathologic factors from the entire dataset.

### Statistical analysis

Baseline characteristics were compared using the independent t test or the Mann–Whitney U test (for continuous variables) and Pearson’s chi-square test or Fisher’s exact test (for categorical variables). Statistical analyses were conducted using SPSS (version 26.0; IBM, Armonk, NY) and R software (version 3.4.4; R Foundation, Vienna, Austria). A two-sided* p* value less than 0.05 indicated a significant difference.

## Results

### Patient characteristics

A total of 214 patients with pathologically proven NPC and IMRT treatment (median age 47.50 years; IQR 37.8–56 years; 69 females) were included, including 135 in the training set and 79 in the validation set. During follow-up, 107 patients were confirmed with RTLI (bilateral, 23; left, 39; right, 45). The median duration of follow-up from the pretreatment MRI was 33.4 months (IQR 26.2–41.9 months) in the RTLI group and 61.4 months (IQR 53.5–68.5 months) in the non-RTLI group. The baseline clinical characteristics are given in Table 1. No significant differences were observed between the training and validation groups (all *p* > 0.05). The rates of RTLI occurrence were 55.56% (75/135) and 40.50% (32/79) in the training and validation cohorts, respectively.

**Table 1:** Characteristics of patients in the training and validation cohorts

Characteristic	Training cohort	Validation cohort	*p* value
	(*n* = 135)	(*n* = 79)	
Age (y)*	46.67 ± 12.81 (9–73)	44.43 ± 13.59 (11–69)	0.23
Sex			
Male	88	57	0.29
Female	47	22	
NLRs (mean ± SD) (range) *	3.22 ± 4.38 (0.43–48.63)	3.43 ± 2.31 (0.90–14.18)	0.70
T stage			0.34
T1	8	4	
T2	8	6	
T3	62	45	
T4	57	24	
N stage			0.53
N0	16	12	
N1	47	28	
N2	51	32	
N3	21	7	
TNM stage			0.22
I	1	1	
II	6	3	
III	56	44	
IV	72	31	
Pathology			0.98
Differentiated	43	25	
Undifferentiated	92	54	
Treatment			0.51
A	17	16	
B	51	24	
C	31	14	
D	19	13	
E	8	4	
F	9	8	
PGTV_NX_ (mean ± SD) (range) (Gy) *	73.51 ± 1.25 (67.72–74.20)	73.72 ± 0.87 (69.96–73.92)	0.20
LD_max_ (mean ± SD) (range) (Gy) *	68.41 ± 5.68 (53.72–78.16)	68.11 ± 5.94 (52.86–86.07)	0.72
RD_max_ (mean ± SD) (range) (Gy) *	68.78 ± 5.28 (55.16–78.57)	68.62 ± 5.67 (55.93–86.07)	0.84

^*^Data are mean ± standard deviation; data in parentheses are range. Treatment: A-Radiotherapy, B-Concurrent Chemoradiotherapy, C-Concurrent Chemoradiotherapy + Targeted Therapy, D-Induction chemotherapy + Concurrent Chemoradiotherapy, E-Induction chemotherapy + Concurrent Chemoradiotherapy + Targeted therapy, F-Radiotherapy + Targeted therapy. *p* > 0.05 suggests no significant difference between the subjects in the two cohorts. LD_max_ = maximum dose of left temporal lobe, NLRs = neutrophil-to-lymphocyte ratios, PGTV_NX_ = planning gross tumor volume included the primary nasopharyngeal tumor or enlarged retropharyngeal nodes, RD_max_ = maximum dose of right temporal lobe, TNM = tumor-node-metastasis

### Temporal lobe segmentation

In assessing the reliability of segmentation, the intra-reader Dice value was 0.981 ± 0.002 (range 0.979–0.982).

### Univariate analysis of histogram parameters

Of the 18 histogram parameters derived from ADC maps, energy, kurtosis, maximum, minimum, range, skewness, and total energy were significant in the univariate analysis for predicting RTLI occurrence in the training cohort (Additional file 1: Table S1).

### Multivariate analysis of histogram parameters

Statistically significant variables in the univariate analysis were included in the multivariate logistic regression analysis. The final best prediction model selected by backward stepwise elimination with Akaike information criteria as the stopping rule included kurtosis (*p* = 0.06), maximum energy (*p* = 0.05), range (*p* = 0.06), and total energy (*p* < 0.001) (Table 2).

**Table 2:** Results of multivariate logistic regression histogram parameters in the training set

Variable	β	SE	Wald	p	OR	95% CI	
						Lower	Upper
Skewness	0.82	1.48	0.31	0.58	2.27	0.13	46.15
Kurtosis	− 0.39	0.21	3.62	0.06*	0.67	0.43	0.99
Energy	6.42E−11	1.10E−10	0.34	0.56	1	1	1
Range	− 0.01	0.01	3.49	0.06*	0.99	0.98	1.00
Minimum	− 0.01	0.01	1.51	0.22	0.99	0.97	1.01
Maximum	0.01	0.01	3.75	0.05*	1.01	1.00	1.02
Total energy	− 1.02E−10	2.81E−11	13.20	< 0.001*	1	1	1

CI = confidence interval, OR = odds ratio, SE = standard error. * indicates significant difference

### Clinical feature selection

The results of the univariate and multivariate logistic analyses for clinical and dosimetric features are presented in Table 3. In the multivariate regression analysis, the T stage was a significant clinical predictor of RTLI.

**Table 3:** Clinical predictive factors according to univariate and multivariate logistic regression in the training set

	Univariate analysis			Multivariate analysis		
	Coefficient	OR (95% CI)	p	Coefficient	OR (95% CI)	p
Age	0.01	1.01 (0.986–1.039)	0.37			
Sex	− 0.15	0.86 (0.152–1.575)	0.69			
NLRs	0.13	1.14 (0.932–1.356)	0.21			
T stage	1.12	3.07 (2.516–3.618)	< 0.001	0.88	2.41 (1.204–5.724)	0.02*
N stage	− 0.18	0.83 (0.450–1.218)	0.35			
TNM stage	0.96	2.60 (1.996–3.211)	0.002	0.18	1.19 (0.450–2.883)	0.70
Pathology	− 0.02	0.98 (0.256–1.714)	0.97			
Treatment	− 0.18	0.83 (0.577–1.085)	0.15			
PGTV_NX_	0.35	1.42 (1.105–1.735)	0.03	0.19	1.21 (0.878–1.771)	0.26
LD_max_	0.07	1.07 (1.006–1.131)	0.04	− 0.02	0.98 (0.898–1.073)	0.70
RD_max_	0.10	1.11 (1.037–1.176)	0.004	0.08	1.08 (0.984–1.194)	0.11

CI = confidence interval, LD_max_ = maximum dose of left temporal lobe, NLRs = neutrophil-to-lymphocyte ratios, OR = odds ratio, PGTV_NX_ = planning gross tumor volume included the primary nasopharyngeal tumor or enlarged retropharyngeal nodes, RD_max_ = maximum dose of right temporal lobe, TNM = tumor-node-metastasis. * indicates significant difference

### Development and validation of predictive models

#### Predictive model derived from ADC map

Based on the results of the multivariate logistic analysis, four histogram parameters were integrated into a Rad-score. The Rad-score was calculated using a linear combination of these histogram parameters based on their respective coefficients. The calculation formula is as follows:

$$\log ({\text{Rad}} - {\text{scrore}}) = 10.34 \pm 0.28 \times {\text{Kurtosis + 0}}{.005} \times {\text{Maximum}} \pm {0}{\text{.004}} \times {\text{Range}} \pm {8}{\text{.28E} - {11}} \times {\text{Total}}\;{\text{Energy}}$$

A difference in Rad-score was present between the RTLI and non-RTLI groups in the training set (median [IQR], 0.97 [0.82–0.99] vs. 0.11 [0.03–0.31]; *p* < 0.001) and confirmed in the validation cohort (median [IQR], 0.95 [0.56–0.99] vs. 0.08 [0.02–0.25]; *p* < 0.001) (Additional file 1: Figure S2). The Rad-score yielded an AUC of 0.95 (95% CI 0.91–0.98) in the training cohort and 0.89 (95% CI 0.81–0.97) in the validation cohort.

### Combination of clinical and histogram findings

**Fig. 2:**
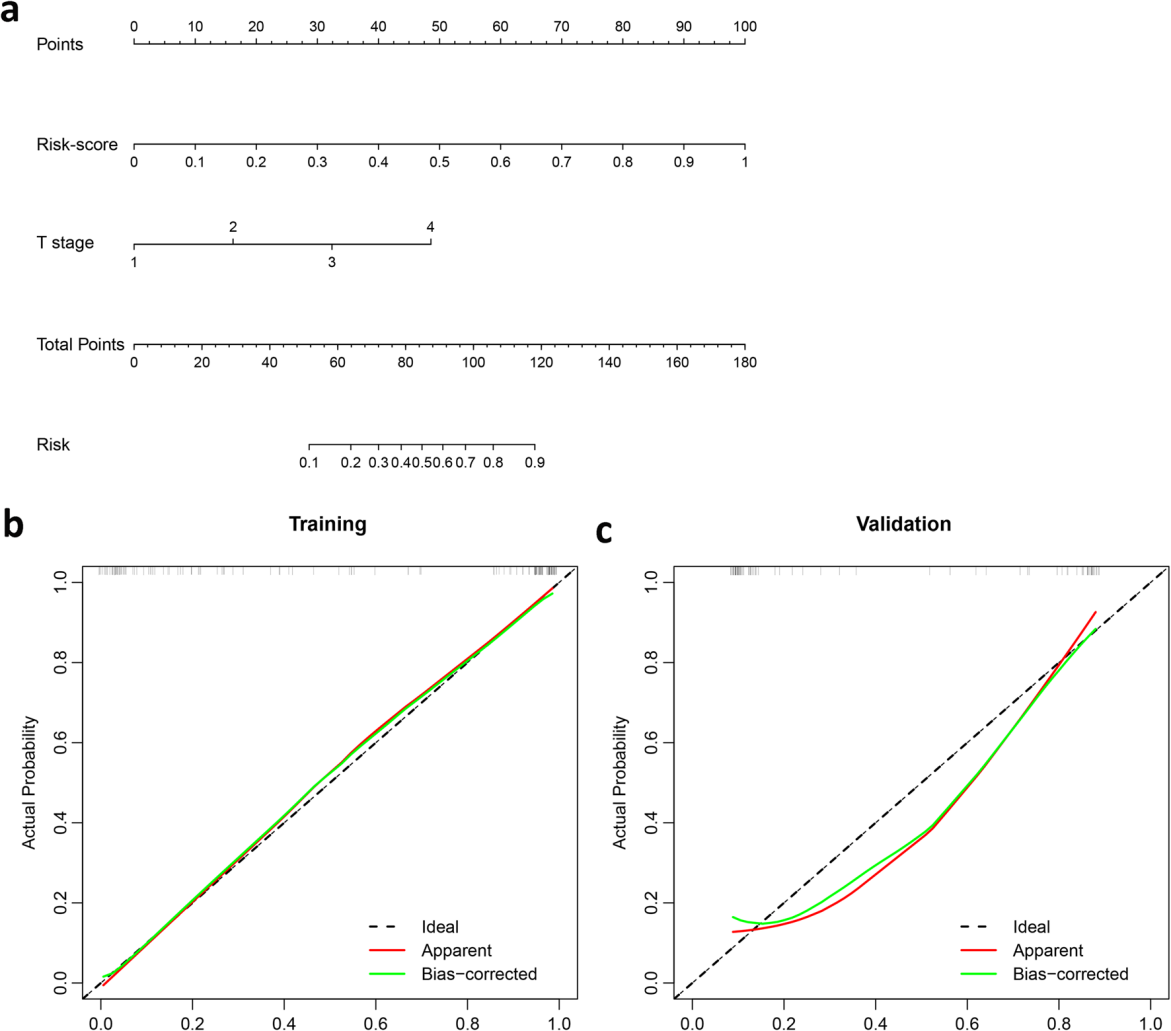
Nomogram and calibration curves. **a** A nomogram was developed in training cohort, with Rad-score and T stage incorporated. Calibration curves of the nomogram in (**b**) training and (**c**) validation cohorts

The variance inflation factors of the five potential predictors ranged from 1.004 to 1.278, indicating no multicollinearity. A combined model incorporating two independent predictors (Rad-score and T stage) was developed and presented as a nomogram (Fig. 2A and Additional file 1: Table S2). The calibration plots showed that the predicted RTLI probabilities of the combined model were in excellent agreement with actual observations (Fig. 2B and C). The Hosmer–Lemeshow test of model calibration showed no departure from a good fit, with no statistical significance (*p* = 0.23).

### Performance and validation of predictive models

**Fig. 3:**
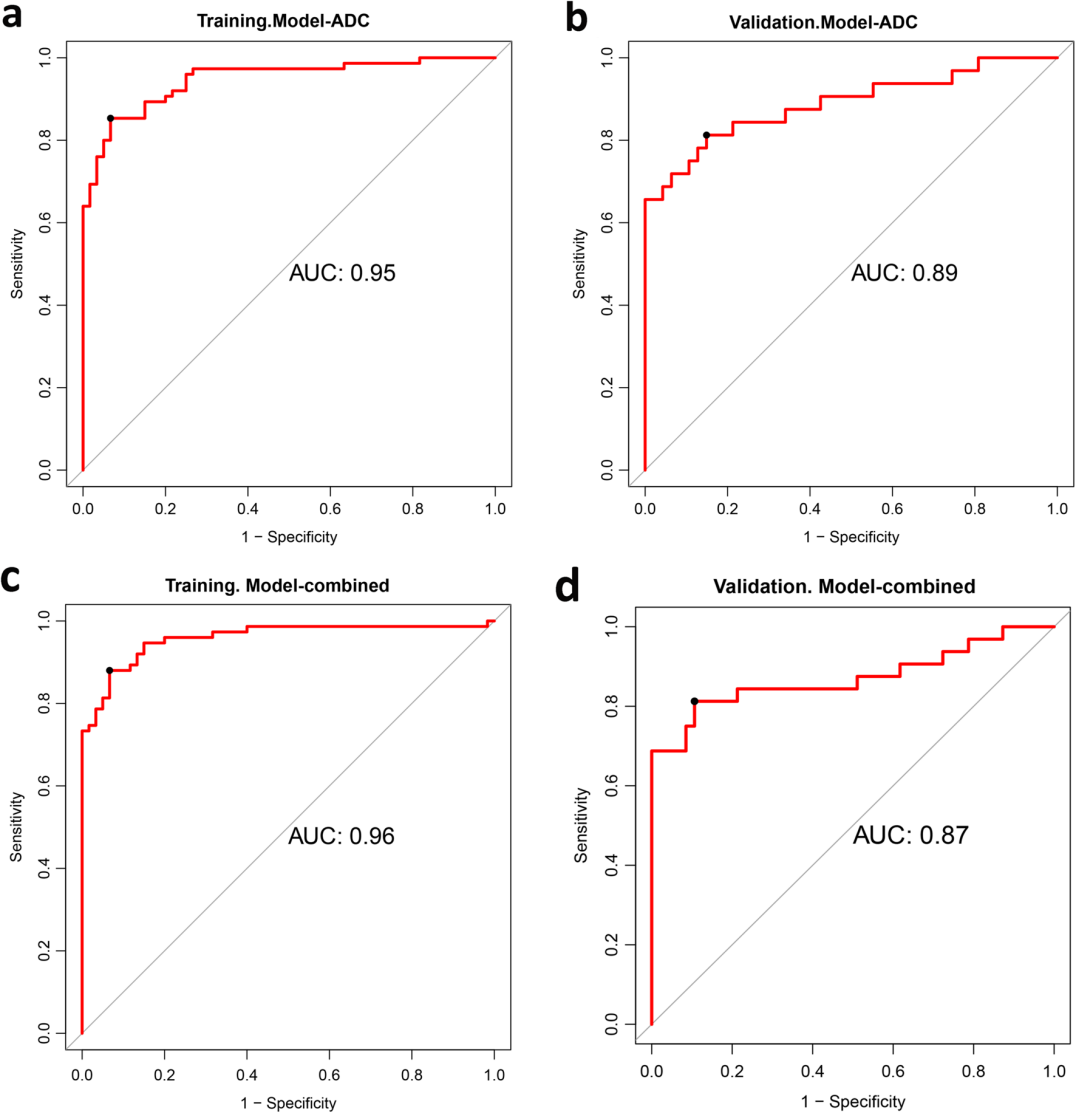
Performances of two models in training cohort and validation cohort, respectively. **a**, **b** Rad-score, including four histogram parameters. **c**, **d** Combined model, integrated T stage and four histogram parameters

The ROC curves of the Rad-score and the combined model are shown in Fig. 3 and Table 4. Compared with the T stage alone (AUC, 0.63 [95% CI 0.52–0.74]), both the Rad-score (*p* < 0.001) and the combined model (*P* < 0.001) exhibited better predictive performance for RTLI after IMRT. The AUC value of the Rad-score (AUC, 0.89) was slightly higher than that of the combined model (AUC, 0.87) in the validation cohort, but the difference was not significant (*p* = 0.47).

**Table 4:** Predictive performances of two models in predicting the radiation-induced temporal lobe injury in the training and validation cohort

Model	AUC	95% CI		Sensitivity	Specificity	PPV	NPV
		Lower	Upper				
Model—ADC							
Training cohort	0.95	0.91	0.98	0.85 (64/75)	0.93 (56/60)	0.94 (64/68)	0.83 (56/67)
Validation cohort	0.89	0.81	0.97	0.75 (24/32)	0.89 (42/47)	0.83 (24/29)	0.89 (42/47)
Model—combined							
Training cohort	0.96	0.92	0.99	0.88 (66/75)	0.93 (56/60)	0.94 (66/70)	0.86 (56/65)
Validation cohort	0.87	0.78	0.96	0.81 (26/32)	0.82 (41/50)	0.81 (26/32)	0.87 (41/47)

AUC = area under the receiver operating characteristic curve, CI = confidence interval, NPV = negative predictive value, PPV = positive predictive value. *Features used for the Model-ADC are kurtosis, maximum, range, and total energy

After obtaining the risk scores based on the combined model, an optimal threshold of 0.55 was determined according to the maximized Youden index from the training cohort. Accordingly, all patients were classified into high- (Rad-score ≥ 0.55) and low-risk (Rad-score < 0.55) RTLI groups (Additional file 1: Figure S3). According to the proposed risk classifier, the combined model achieved a sensitivity of 81.3% and a specificity of 82.0% for predicting RTLI in the validation cohort, whereas the positive and negative predictive values were 81.3% and 87.2%, respectively. Moreover, when the patients were stratified based on clinicopathological factors, the overall diagnostic accuracy of the risk classifier was excellent in all subgroups (AUC, 0.79–0.98). The performance of the constructed combined model in patients within different clinicopathological subgroups is presented in Additional file 1: Figure S4.

Additionally, DCA indicated that the Rad-score or combined model achieved moderately better net benefits than clinical factors alone (Additional file 1: Figure S5). The positive values of integrated discrimination improvement (53.0% [95% CI 0.45–0.61], *p* < 0.001) and net reclassification index (69.0% [95% CI 0.55–0.83], *p* < 0.001) are shown.

## Discussion

In this study, we assessed the capability of pretreatment histogram parameters in predicting RTLI in patients with NPC after IMRT. Our results showed that the Rad-score integrating the four histogram parameters was an independent predictive factor of RTLI and showed a favorable predictive performance. A nomogram combining T stage and Rad-score as a quantitative tool could facilitate RTLI risk stratification and clinical decision-making in NPC patients treated with IMRT.

While histogram analysis has been successfully demonstrated in various organs [33–35] and the predictive potential of radiomics features has been explored in RTLI in NPC patients [21, 22, 24, 25], the utility of histogram parameters in predicting RTLI still needs to be further investigated. As previously suggested, image heterogeneity is correlated with physiological heterogeneity [20]. We found that some of the histogram parameters derived from ADC mapping of the temporal lobes were associated with RTLI occurrence. Kurtosis yielded the highest (negative) coefficient in selected histogram parameters, which is a measure of the “peakedness” of the distribution of values in the image ROI [36]. A lower kurtosis implies that the mass of the distribution is concentrated toward a spike near the mean value, implying that the temporal lobes were more functionally homogeneous. The range represents the range of gray values in the voxel of interest (VOI), whereas total energy refers to the value of the energy feature scaled by the volume of the voxel in mm^3^ [36]. Higher values of range and total energy may indicate the complexity of the tissue components. In this study, lower values of kurtosis, range, and total energy, which led to a higher Rad-score, were associated with patients more prone to developing RTLI. As no histological proof of the precise mechanism that leads to RTLI and its association with the heterogeneity of temporal lobes was available by this point, we can hypothesize that less heterogeneous image textures corresponded to the abundance of cells in the VOI of the temporal lobe, with the cells arranged tightly and regularly [37, 38]. Furthermore, the abundant blood supply and high oxygen demand of the corresponding temporal lobe, which means greater sensitivity to radiotherapy [39, 40], were more prone to developing RTLI. It is well known that a high cell density is associated with a low ADC [41, 42], and the region showing the maximum ADC may reflect the lowest cellular area within the temporal lobe. However, the results of our study indicate that the maximum value was positively correlated with RTLI occurrence, which contradicts our previous hypothesis. It is difficult to provide a reasonable explanation based on the current research, and further research on the histological proof of temporal lobe heterogeneity and RTLI occurrence is required.

Compared with other studies that established prediction models for predicting RTLI based on pretreatment MRI parameters, the AUC of the model in this study was lower than that of the prediction model based on radiomics features extracted from contrast-enhanced T1- or fat-suppressed T2-weighted MRI (AUC, 0.89 vs. 0.92) [24], and higher than that of the proposed model based on features extracted from T1- and T2-weighted MRI (AUC, 0.82) [25]. Therefore, the prediction model based on ADC histogram parameters showed persuasive performance in predicting RTLI in NPC, and the feasibility of a multiparametric MRI model to predict RTLI should be explored in future studies.

Concerning clinical predictors, the T stage was identified as a clinical predictor for RTLI in our study, which was consistent with the findings of Wen et al. [9] and Guan et al. [8]. This study demonstrated that the nomogram incorporating histogram parameters and T stage yielded satisfactory predictive performance, with favorable calibration and positive net reclassification improvement. DCA also illustrated that both the combined model and the Rad-score outperformed the T stage alone in predicting RTLI occurrence, but interestingly, the combined model did not significantly improve the predictive performance compared to the Rad-score; a similar lack of improvement in the extended model compared with individual components has been previously observed in brain tumors [43] and is attributed to the high intra-correlation of ADC histogram parameters.

This study had several limitations. First, selection bias exists because of the retrospective design of the study. Removing a significant portion of patients for a variety of reasons may have generated bias. Second, patients without RTLI after IMRT were included by a propensity score matching at 1:1 to each RTLI case by gender in this study. Third, patients without RTLI after IMRT were included by propensity score matching at 1:1 for each RTLI case by sex in this study. The preferred design should include all patients to ensure that no bias is introduced for all relevant risk factors and outcomes; however, the low incidence of RTLI in the clinic and the long follow-up time needed for RTLI outcomes in NPC may make the research difficult to implement. Fourth, we performed DWI using only two *b* values of 0 and 800 s/mm^2^ on a 3.0-T MRI machine from a single manufacturer; further studies on DWI with multiple b values with various MRI scanners and techniques may contribute to the generalizability of the results. Finally, the dosimetric parameters included in this study were limited and not independent predictors of RTLI in the training set; therefore, we did not include them in the final prediction model. Although patients with NPC who received radiotherapy were one of the causative factors for possible RTLI, patients included in this study were treated with IMRT and standardized treatment according to their conditions. Therefore, in this case, the predictive model still had predictive potential for RTLI in patients with NPC who received IMRT. The prediction model used in this study was based on MRI obtained before treatment. Patients receiving radiotherapy may have subtle changes that are invisible to the naked eye and can be detected early using radiomics. Prediction models based on MRI obtained immediately after IMRT may yield different results [21, 22, 24]. In general, the feasibility of histograms, clinical and dosimetric parameters, white and gray matter, and their associated variables were considered separately, and MRI after IMRT to predict RTLI should be explored in future studies, especially prospective studies with larger sample sizes at multicenter institutions.

## Conclusions

In summary, our study revealed that histogram parameters of ADC mapping based on temporal lobes are related to RTLI occurrence in patients with NPC after IMRT. The combined model achieved satisfactory pretreatment risk prediction of RTLI in patients with NPC, which may help stratify high-risk patients who require intensive follow-up and effective treatment guidance.

## Supplementary Information

**Additional file 1.** Supplementary tables and figures.

## Data Availability

The datasets used and/or analyzed during the current study are available from the corresponding author upon reasonable request.

## Abbreviations

ADC: Apparent diffusion coefficient
AUC: Area under the curve
CI: Confidence interval
DCA: Decision curve analysis
DTI: Diffusion tensor imaging
DWI: Diffusion-weighted imaging
IMRT: Intensity-modulated radiotherapy
IQR: Interquartile range
MRI: Magnetic resonance imaging
NPC: Nasopharyngeal carcinoma
ROC: Receiver operating characteristic
ROI: Region of interest
RTLI: Radiation-induced temporal lobe injury
TLI: Temporal lobe injury
VOI: Voxel of interest

## References

[1] Chen YP, Chan ATC, Le QT, Blanchard P, Sun Y, Ma J (2019). Nasopharyngeal carcinoma. Lancet.

[2] Greene-Schloesser D, Robbins ME, Peiffer AM, Shaw EG, Wheeler KT, Chan MD (2012). Radiation-induced brain injury: a review. Front Oncol.

[3] Tang Y, Luo D, Rong X, Shi X, Peng Y (2012). Psychological disorders, cognitive dysfunction and quality of life in nasopharyngeal carcinoma patients with radiation-induced brain injury. PLoS One.

[4] Lee N, Harris J, Garden AS (2009). Intensity-modulated radiation therapy with or without chemotherapy for nasopharyngeal carcinoma: radiation therapy oncology group phase II trial 0225. J Clin Oncol.

[5] Zhou GQ, Yu XL, Chen M (2013). Radiation-induced temporal lobe injury for nasopharyngeal carcinoma: a comparison of intensity-modulated radiotherapy and conventional two-dimensional radiotherapy. PLoS One.

[6] Liang SB, Wang Y, Hu XF (2017). Survival and toxicities of IMRT based on the RTOG protocols in patients with nasopharyngeal carcinoma from the endemic regions of China. J Cancer.

[7] Abayomi OK (2002). Pathogenesis of cognitive decline following therapeutic irradiation for head and neck tumors. Acta Oncol.

[8] Guan W, Xie K, Fan Y (2020). Development and validation of a nomogram for predicting radiation-induced temporal lobe injury in nasopharyngeal carcinoma. Front Oncol.

[9] Wen DW, Lin L, Mao YP (2021). Normal tissue complication probability (NTCP) models for predicting temporal lobe injury after intensity-modulated radiotherapy in nasopharyngeal carcinoma: a large registry-based retrospective study from China. Radiother Oncol.

[10] Huang J, Kong FF, Oei RW, Zhai RP, Hu CS, Ying HM (2019). Dosimetric predictors of temporal lobe injury after intensity-modulated radiotherapy for T4 nasopharyngeal carcinoma: a competing risk study. Radiat Oncol.

[11] Feng M, Huang Y, Fan X, Xu P, Lang J, Wang D (2018). Prognostic variables for temporal lobe injury after intensity modulated-radiotherapy of nasopharyngeal carcinoma. Cancer Med.

[12] Lee AW, Cheng LO, Ng SH (1990). Magnetic resonance imaging in the clinical diagnosis of late temporal lobe necrosis following radiotherapy for nasopharyngeal carcinoma. Clin Radiol.

[13] Padhani AR, Liu G, Koh DM (2009). Diffusion-weighted magnetic resonance imaging as a cancer biomarker: consensus and recommendations. Neoplasia.

[14] Ota Y, Liao E, Kurokawa R (2021). Diffusion-weighted and dynamic contrast-enhanced MRI to assess radiation therapy response for head and neck paragangliomas. J Neuroimaging.

[15] Ota Y, Liao E, Capizzano AA (2021). Diagnostic role of diffusion-weighted and dynamic contrast-enhanced perfusion MR imaging in paragangliomas and schwannomas in the head and neck. AJNR Am J Neuroradiol.

[16] Martens RM, Stappen RV, Koopman T et al (2020) The additional value of ultrafast DCE-MRI to DWI-MRI and 18F-FDG-PET to detect occult primary head and neck squamous cell carcinoma. Cancers (Basel) 12 10.3390/cancers12102826 PMC7600235 33007978

[17] Martens RM, Koopman T, Lavini C (2021). Multiparametric functional MRI and (18)F-FDG-PET for survival prediction in patients with head and neck squamous cell carcinoma treated with (chemo)radiation. Eur Radiol.

[18] Koontz NA, Wiggins RH (2017). Differentiation of benign and malignant head and neck lesions with diffusion tensor imaging and DWI. AJR Am J Roentgenol.

[19] Liu X, Han C, Wang H (2021). Fully automated pelvic bone segmentation in multiparameteric MRI using a 3D convolutional neural network. Insights Imaging.

[20] Just N (2014). Improving tumour heterogeneity MRI assessment with histograms. Br J Cancer.

[21] Zhang B, Lian Z, Zhong L (2020). Machine-learning based MRI radiomics models for early detection of radiation-induced brain injury in nasopharyngeal carcinoma. BMC Cancer.

[22] Hou J, Li H, Zeng B (2022). MRI-based radiomics nomogram for predicting temporal lobe injury after radiotherapy in nasopharyngeal carcinoma. Eur Radiol.

[23] Wang HZ, Qiu SJ, Lv XF (2012). Diffusion tensor imaging and 1H-MRS study on radiation-induced brain injury after nasopharyngeal carcinoma radiotherapy. Clin Radiol.

[24] Bao D, Zhao Y, Li L (2022). A MRI-based radiomics model predicting radiation-induced temporal lobe injury in nasopharyngeal carcinoma. Eur Radiol.

[25] Bin X, Zhu C, Tang Y (2022). Nomogram based on clinical and radiomics data for predicting radiation-induced temporal lobe injury in patients with non-metastatic stage T4 nasopharyngeal carcinoma. Clin Oncol (R Coll Radiol).

[26] Adelstein D, Gillison ML, Pfister DG (2017). NCCN guidelines insights: head and neck cancers, version 2.2017. J Natl Compr Canc Netw.

[27] Ren W, Liang B, Sun C (2021). Dosiomics-based prediction of radiation-induced hypothyroidism in nasopharyngeal carcinoma patients. Phys Med.

[28] Wang YX, King AD, Zhou H (2010). Evolution of radiation-induced brain injury: MR imaging-based study. Radiology.

[29] Duane F, Aznar MC, Bartlett F (2017). A cardiac contouring atlas for radiotherapy. Radiother Oncol.

[30] Moons KG, Altman DG, Reitsma JB (2015). Transparent Reporting of a multivariable prediction model for individual prognosis or diagnosis (TRIPOD): explanation and elaboration. Ann Intern Med.

[31] Sauerbrei W, Boulesteix AL, Binder H (2011). Stability investigations of multivariable regression models derived from low- and high-dimensional data. J Biopharm Stat.

[32] Kim JH (2019). Multicollinearity and misleading statistical results. Korean J Anesthesiol.

[33] Perucho JAU, Wang M, Tse KY (2021). Association between MRI histogram features and treatment response in locally advanced cervical cancer treated by chemoradiotherapy. Eur Radiol.

[34] Wu LF, Rao SX, Xu PJ (2019). Pre-TACE kurtosis of ADCtotal derived from histogram analysis for diffusion-weighted imaging is the best independent predictor of prognosis in hepatocellular carcinoma. Eur Radiol.

[35] Kang Y, Choi SH, Kim YJ (2011). Gliomas: Histogram analysis of apparent diffusion coefficient maps with standard- or high-b-value diffusion-weighted MR imaging–correlation with tumor grade. Radiology.

[36] van Griethuysen JJM, Fedorov A, Parmar C (2017). Computational radiomics system to decode the radiographic phenotype. Cancer Res.

[37] Yang J, Xu Z, Gao J (2018). Evaluation of early acute radiation-induced brain injury: hybrid multifunctional MRI-based study. Magn Reson Imaging.

[38] Bashir U, Foot O, Wise O (2018). Investigating the histopathologic correlates of 18F-FDG PET heterogeneity in non-small-cell lung cancer. Nucl Med Commun.

[39] Jia Y, Weng Z, Wang C (2017). Increased chemosensitivity and radiosensitivity of human breast cancer cell lines treated with novel functionalized single-walled carbon nanotubes. Oncol Lett.

[40] Xie Y, Huang H, Guo J, Zhou D (2018). Relative cerebral blood volume is a potential biomarker in late delayed radiation-induced brain injury. J Magn Reson Imaging.

[41] Sugahara T, Korogi Y, Kochi M (1999). Usefulness of diffusion-weighted MRI with echo-planar technique in the evaluation of cellularity in gliomas. J Magn Reson Imaging.

[42] Chen J, Xia J, Zhou YC (2005). Correlation between magnetic resonance diffusion weighted imaging and cell density in astrocytoma. Zhonghua Zhong Liu Za Zhi.

[43] Kyriazi S, Collins DJ, Messiou C (2011). Metastatic ovarian and primary peritoneal cancer: assessing chemotherapy response with diffusion-weighted MR imaging–value of histogram analysis of apparent diffusion coefficients. Radiology.

