# Supplementary material for: Added value of histogram analysis of ADC in predicting radiation-induced temporal lobe injury of patients with nasopharyngeal carcinoma treated by intensity-modulated radiotherapy
Source: Insights Imaging. 2022 Dec 17;13:197. doi: 10.1186/s13244-022-01338-w (PMC9759610; doi:10.1186/s13244-022-01338-w)

ELECTRONIC SUPPLEMENTARY MATERIAL

Added-value of histogram analysis of ADC in predicting radiation-induced temporal lobe injury of patients with nasopharyngeal carcinoma treated by intensity-modulated radiotherapy

Table S1. Results of univariate logistic regression histogram parameters in the training set

| **Variable** | **β** | **SE** | **Wald** | **p** | **OR** | **95%CI** | |
| --- | --- | --- | --- | --- | --- | --- | --- |
|  |  |  |  |  |  | **lower** | **upper** |
| **Skewness** | -0.83 | 0.35 | 5.59 | 0.02* | 0.43 | -0.25 | 1.12 |
| **Kurtosis** | -0.15 | 0.05 | 7.93 | 0.005* | 0.86 | 0.76 | 0.96 |
| **Entropy** | -0.19 | 0.38 | 0.26 | 0.61 | 0.82 | 0.07 | 1.57 |
| **Energy** | -2.93E- 10 | 4.82E-11 | 36.90 | <0.001* | 1 | 1 | 1 |
| **Range** | -0.0009 | 0.0002 | 14.58 | <0.001* | 0.99 | 0.99 | 0.99 |
| **Uniformity** | 3.89 | 11.12 | 0.12 | 0.73 | 48.93 | 27.13 | 70.73 |
| **Mean** | -0.002 | 0.003 | 0.58 | 0.45 | 0.99 | 0.99 | 1.00 |
| **Median** | -0.001 | 0.003 | 0.16 | 0.69 | 0.99 | 0.99 | 1.00 |
| **Minimum** | 0.007 | 0.002 | 15.87 | <0.001* | 1.01 | 1.00 | 1.01 |
| **Maximum** | -0.0007 | 0.0003 | 7.83 | 0.005* | 0.99 | 0.99 | 0.99 |
| **Variance** | -2.61E- 06 | 4.52E-06 | 0.33 | 0.56 | 0.99 | 0.99 | 1.00 |
| **10th**  **percentile** | -0.001 | 0.002 | 0.38 | 0.54 | 0.99 | 0.99 | 1.00 |
| **90th**  **percentile** | -0.0004 | 0.001 | 0.10 | 0.75 | 0.99 | 0.99 | 1.00 |
| **IQR** | 0.0001 | 0.002 | 0.002 | 0.96 | 1.00 | 0.99 | 1.00 |
| **MAD** | -0.001 | 0.003 | 0.10 | 0.75 | 0.99 | 0.99 | 1.00 |
| **RMAD** | 0.0004 | 0.005 | 0.006 | 0.94 | 1.00 | 0.99 | 1.01 |
| **RMS** | -0.002 | 0.002 | 0.62 | 0.43 | 0.99 | 0.99 | 1.00 |
| **Total-energy** | -7.49E- 11 | 1.21E-11 | 38.30 | <0.001* | 1 | 1 | 1 |

CI = confidence interval, IQR = interquartile range, MAD = mean absolute deviation, OR = odds ratio, RMAD = robust mean absolute deviation, RMS = root mean squared, SE = standard error. * indicates risk factors included in the multivariate logistic regression analysis.

Table S2. Multivariable Logistic regression analysis of Risk-score and T stage in the training cohort

| **Variable** | **β** | **SE** | **Wald** | **p** | **OR** | **95%CI** | |
| --- | --- | --- | --- | --- | --- | --- | --- |
|  |  |  |  |  |  | **lower** | **upper** |
| T stage | 1.07 | 0.41 | 6.64 | 0.01* | 2.92 | 1.34 | 6.97 |
| Rad-Score | 6.61 | 1.04 | 40.24 | <0.001* | 741.99 | 120.66 | 7696.95 |

Note.—Data are results of the multivariable regression analysis. CI = confidence interval, OR = odds ratio, SE = standard error. * indicates significant difference.

**Supplementary Figure S1.** Temporal lobe segmentation. (a) Temporal lobe ROIs was drawn on the b = 800 s/mm^2^ DWI of the pretreatment MRI . (b) The ROIs were propagated to ADC maps.


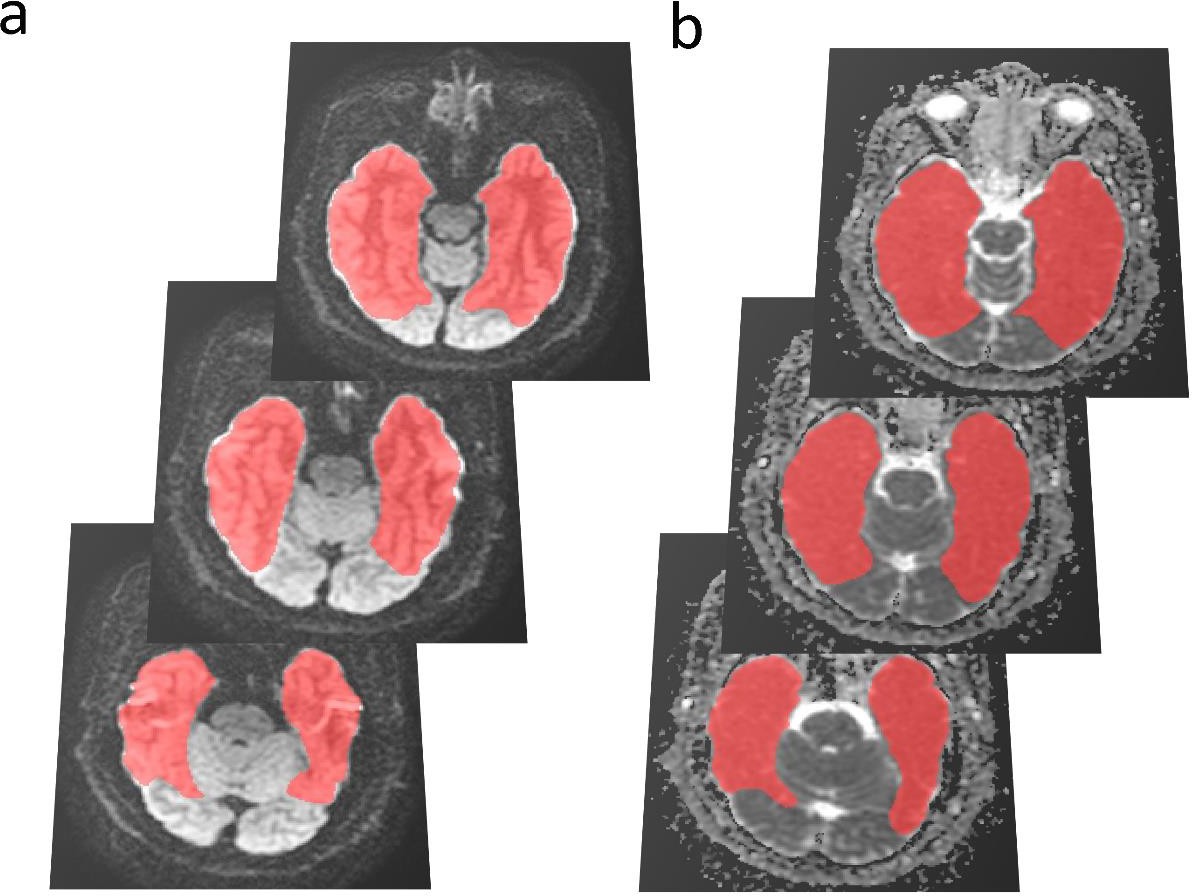


**Supplementary Figure S2.** Radiomics score between patients with and without radiation-induced temporal lobe injury.

RTLI = radiation-induced temporal lobe injury.


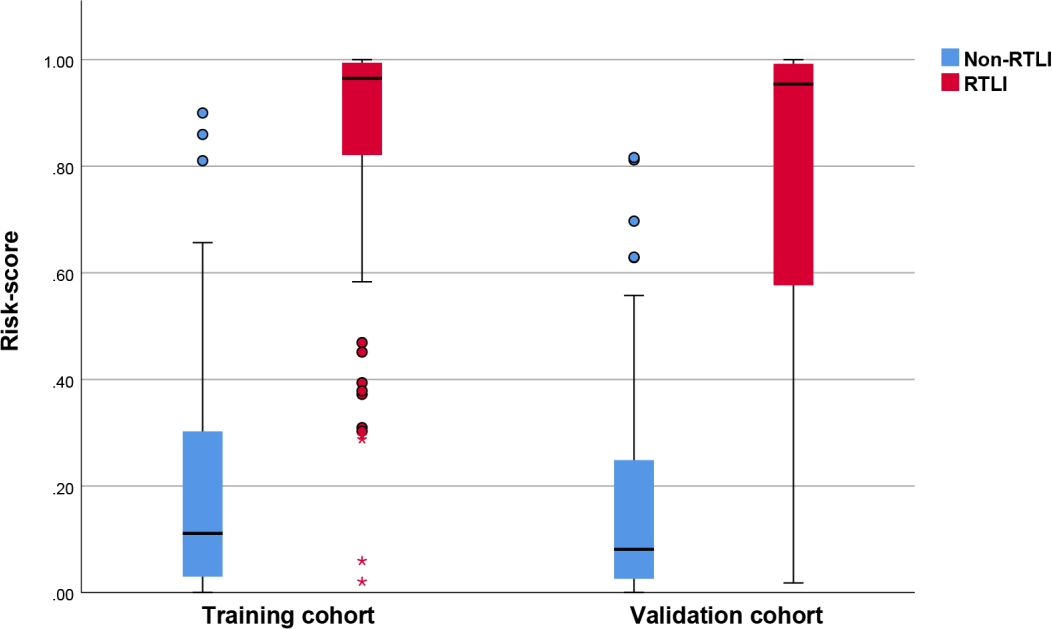


**Supplementary Figure S3.** Risk score for every patient by the combined model-based classifier in training cohort, validation cohort respectively.

RTLI = radiation-induced temporal lobe injury.


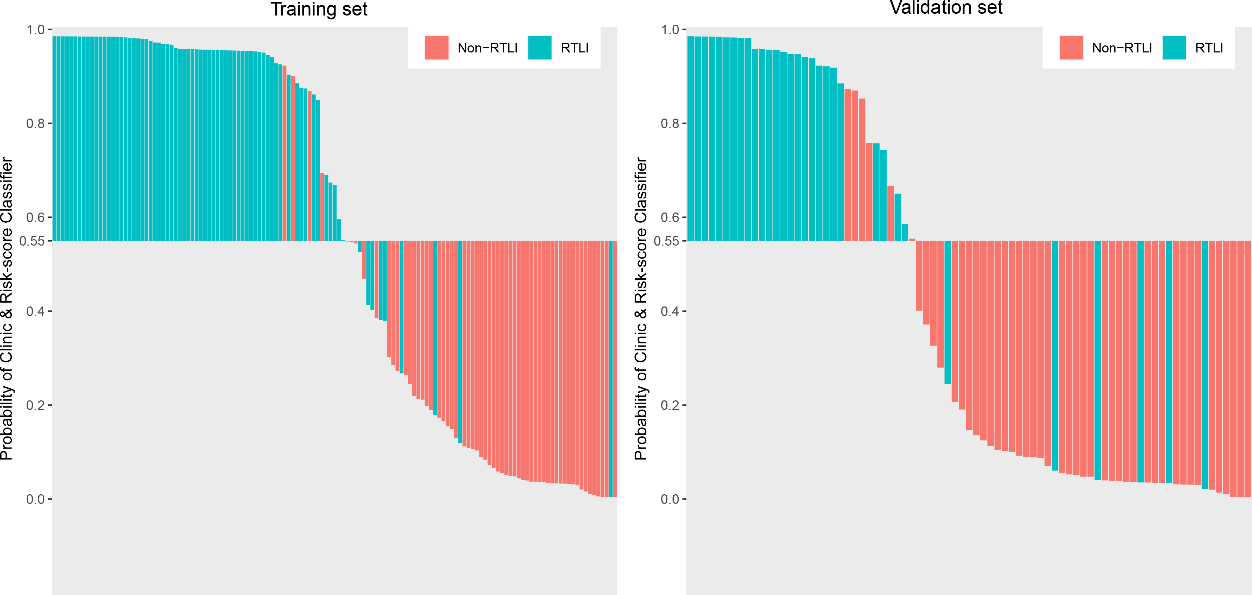


**Supplementary Figure S4.** The performances of the combined model within different clinical-pathologic subgroups. (a, b) gender (male or female), (c, d) age (<40 or ≥40), (e, f) TNM stage (I-III or IV), (g, h) pathologic type (differentiated non- keratinizing, or undifferentiated non-keratinizing), (i, j) Dmax of left temporal lobe (<68 Gy or ≥68 Gy), and (k, l) Dmax of right temporal lobe (<68 Gy or ≥68 Gy). AUC = area under the curve, LDmax = maximum dose for left temporal lobe, RDmax = maximum dose for right temporal lobe.


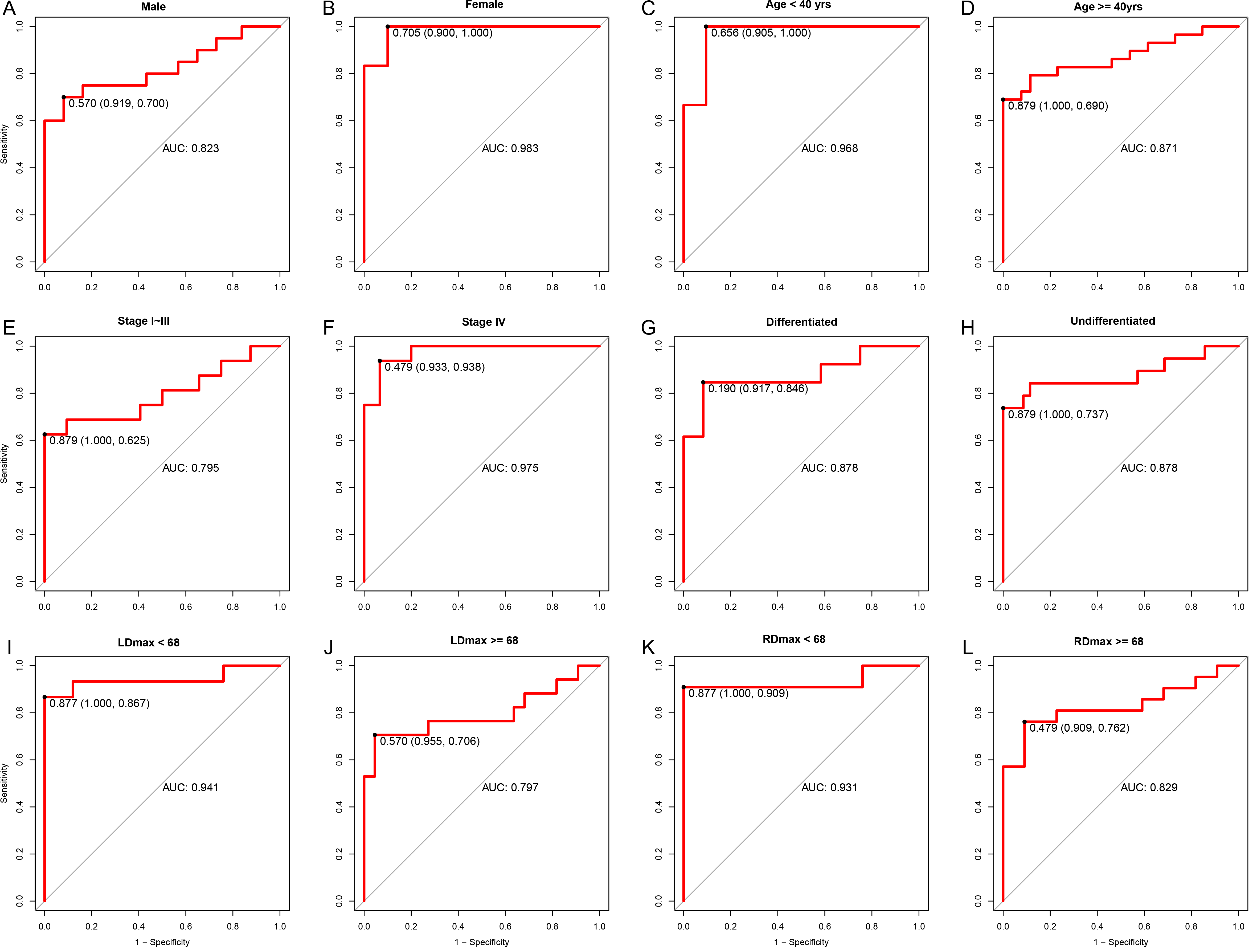


**Supplementary Figure S5.** Decision curve analysis for each model in validation dataset. The y-axis measures the net benefit, which is calculated by summing the benefits (true-positive findings) and subtracting the harms (false-positive findings), weighting the latter by a factor related to the relative harm of undetected radiation-induced temporal lobe injury (RTLI) compared with the harm of unnecessary treatment.


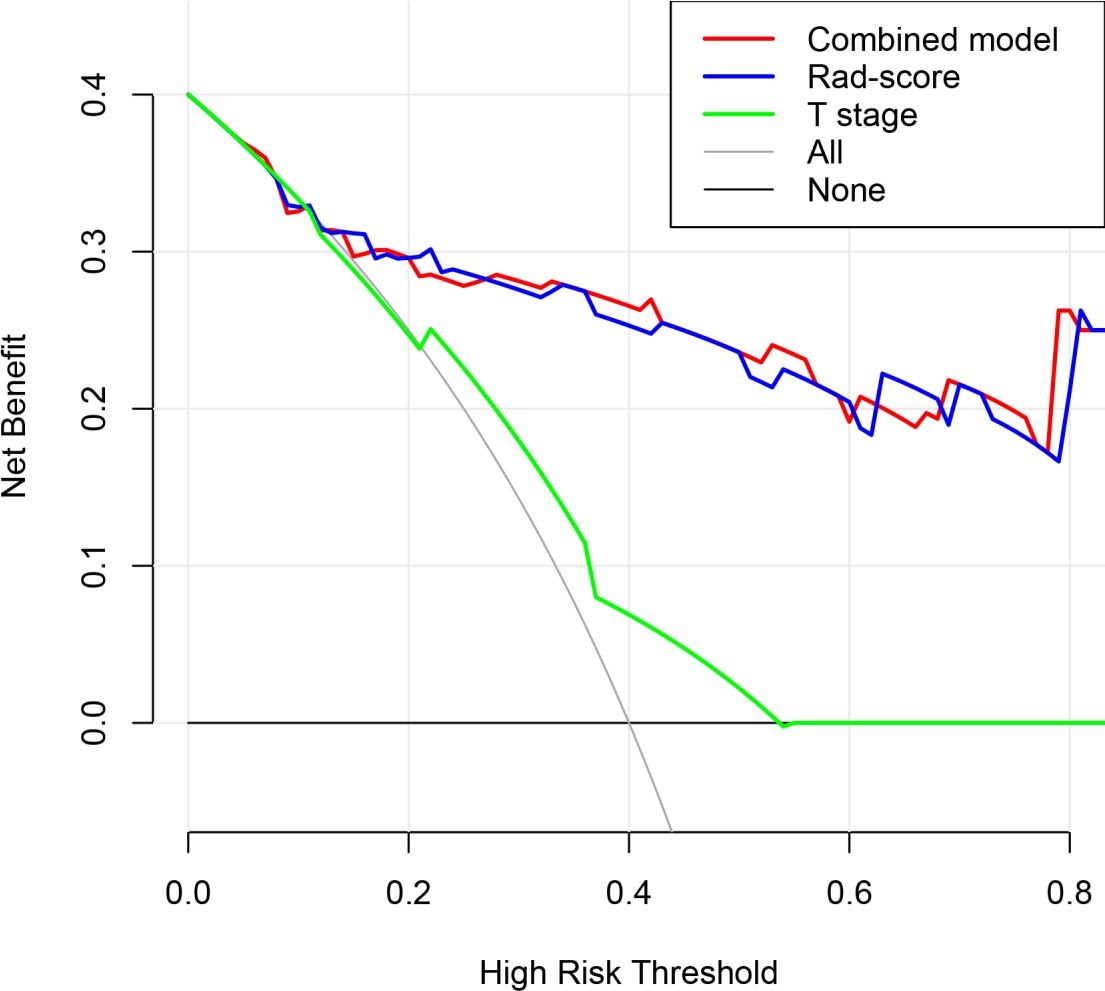

Supplement: Supplementary file 1 — Additional file 1. Supplementary tables and figures. [file 13244_2022_1338_MOESM1_ESM.docx]
